# Dental students’ self-evaluation comparison between dual dental education systems in Korea

**DOI:** 10.1186/s12909-022-03504-6

**Published:** 2022-06-06

**Authors:** Young-A Ji, Yang-Jo Seol, Jungjoon Ihm

**Affiliations:** 1 Dental Research Institute, School of Dentistry, School of Dentistry, Seoul National University, Seoul, Republic of Korea; 2 Department of Periodontology, School of Dentistry, Seoul National University, Seoul, Republic of Korea; 3 Department of Dental Education, School of Dentistry, Seoul National University, Seoul, Republic of Korea

**Keywords:** Dental education system, Self-evaluation, Dental education

## Abstract

**Background:**

This study aimed to examine the satisfaction, educational linkage, and self-perceived competence of dental students enrolled in either 4 + 4 dental program, comprising an undergraduate degree and Doctor of Dental Surgery degree (DDS), or 3 + 4 program, which is a BS/DDS combined degree program, in the Korean dental education system.

**Materials and methods:**

The survey questionnaire using a 5-point Likert scale was developed and validated by four dental education experts, consisting of satisfaction with undergraduate courses, the interconnection of undergraduate courses with the DDS curriculum, and self-assessed core competency for dental graduates. A total of 252 students provided informed consent and voluntarily responded to the survey, among whom 109 students were in the 3 + 4 system and 143 were in the 4 + 4 system. Cronbach’s correlation analysis and independent t-test were conducted for each evaluation item.

**Results:**

Students’ overall satisfaction level with the undergraduate education was higher in the 4 + 4 system than in the 3 + 4 system (*P* = 0.003). Students enrolled in the 4 + 4 system recognized that natural sciences are more connected to the graduate-level DDS program (*P* <  0.001), while the 3 + 4 students recognized that studies in medicine are closely interconnected to the DDS program (*P* = 0.001). There was almost no statistically significant difference in the students’ perception of competency between the two education systems.

**Discussion:**

Even though this study analyzed the case of a single university operating both 3 + 4 and 4 + 4 systems, it can be used as the groundwork for developing new opportunities and models of dental education system.

**Supplementary Information:**

The online version contains supplementary material available at 10.1186/s12909-022-03504-6.

## Introduction

In compliance with the changes and demands of society, the educational environment of colleges and graduate-entry schools of dentistry are facing diverse transitions. To achieve successful educational performance, one of the most important challenges for the dental education environment is to improve the level of student satisfaction with their dental school and the quality of education they receive [1]. Korea has announced an introductory model of dental education for the reorganization and improvement of medical and dental education.

There are a variety of dental education systems worldwide: 4 + 4 or 4 + 3 system in the United States (US), 4 to 5 years in the UK, 5 to 6 years in China, 6-year system in Japan and Hong Kong, and 5-year system in some European countries [2, 3]. As of 2022, 68 universities in the US operate dental schools, most of which operate a 4 + 4 system. Some universities offer integrated curriculum and multidisciplinary programs as they attempt to develop different systems of dental education.

Prior to the year 2000, the curriculum of Korean dental colleges was completed within a single six-year system, composed of 2 + 4 system, where the first 2 years of pre-dentistry focused on liberal arts and basic science and the latter 4 years included professional DDS coursework [3]. Students were required to take the national standardized test in their senior year of high school, and they received a DDS degree at the end of 2 + 4 system.

In the midst of complicated and diverse social demands and changes, Korean dental schools have maintained a mixture of education systems throughout the 2000s. In particular, the transition was prompted by the inflexibility of the 2 + 4 system in academic fields that guarantee a career as a professional, such as dental and medical programs. As students enter these programs immediately after high school and cannot change their major once enrolled, they may have an inadequate understanding of interdisciplinary insight, which ultimately hinders the development of the field itself.

After 2003, 4-year college graduates with BA or BS degrees were allowed to enter a newly created 4-year DDS program at graduate school in dentistry, switching the traditional 2 + 4 coursework into 4 + 4 professional graduate programs to train professionals specialized in dentistry, similar to the current dental education programs found in the US [4]. The new 4 + 4 dental education system, also called the graduate-entry dental school program, combines the 4 years of undergraduate study (bachelor’s degree) at a different institution with the 4 years of dental study at the graduate-level at a school of dentistry.

**Fig. 1:**
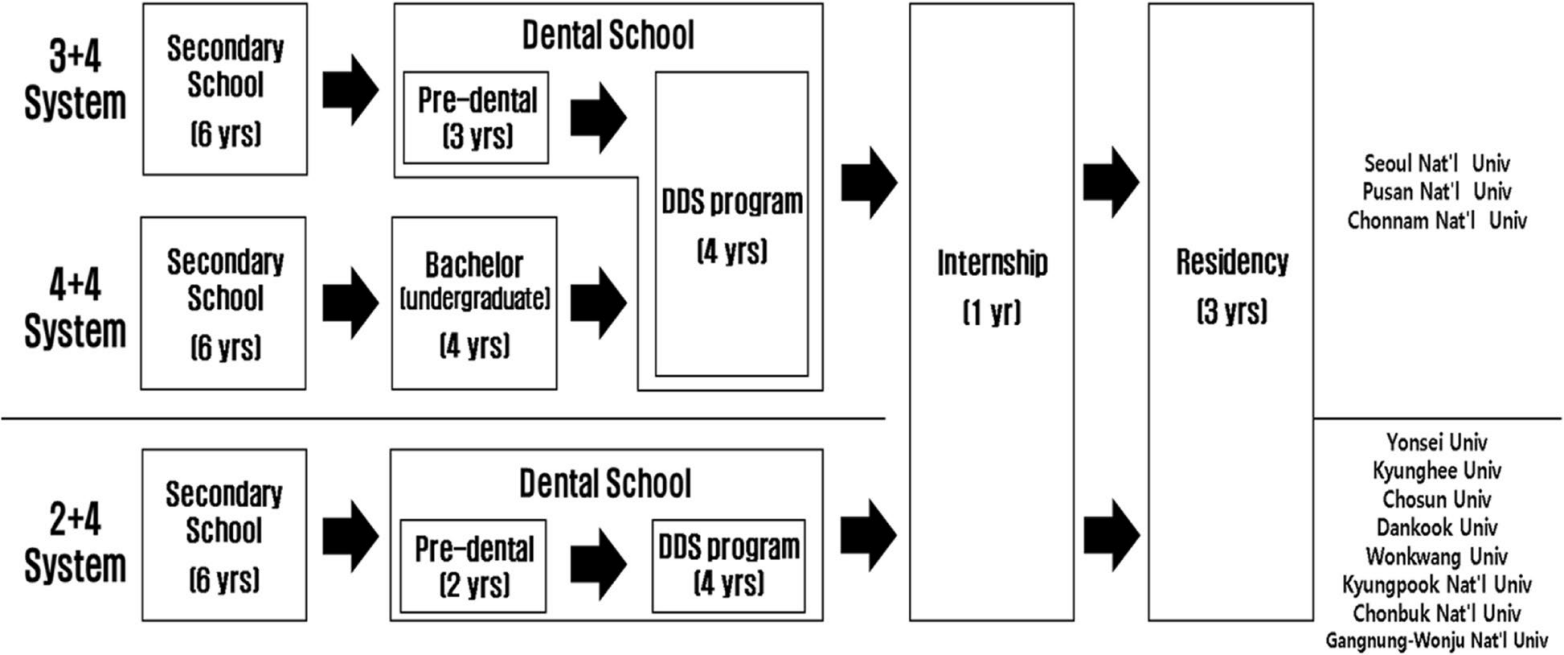
Comparison of different dental education systems in Korea

In addition to the 4 + 4 system, the 3 + 4 system was introduced as a combination program, composed of a three-year pre-dental undergraduate curriculum and a four-year DDS graduate curriculum. As of now, three of the 11 dental schools in Korea operate both 3 + 4 and 4 + 4 programs. However, the remaining eight dental schools have chosen to maintain the traditional 2 + 4 programs (Fig. 1). The 3 + 4 system is unique in that it has not been attempted in any overseas dental program. Therefore, this study was conducted to grasp the educational satisfaction of students and compare student evaluations of the two systems in a university currently operating 3 + 4 and 4 + 4 systems together. Indeed, educational satisfaction assessments conducted on students are considered to be the most reliable measure of obtaining information about the curriculum [5, 6].

When it comes to dental school admission by school system in Korea, any candidates applying for undergraduate college including 2 + 4 or 3 + 4 systems must take the College Scholastic Ability Test, which is similar to the American Scholastic Aptitude Test. Test scores, along with the high school grade point average, recommendations, and essay are submitted to the admissions office. Candidates applying for admission to four-year dental education programs with a 4 + 4 system are required to have a BA/BS degree of any major and take Dental Education Eligibility Test, which similar to Dental Admissions Test in the US. In addition, candidates are often required to make certain grades on certified English proficiency test and undergo an oral examination and interviews. Although any major can apply for dental school, pre-dentistry courses in natural science, humanities, and sociology are required.

According to each school system, different degrees are awarded from Korean dental schools. Upon completion of the 2 + 4 system, students receive an undergraduate degree level DDS, whereas students completing the 3 + 4 curriculum receive the same graduate degree level DDS as those in the 4 + 4 system. The only difference between the undergraduate and graduate degree levels is whether students can continue graduate study within the dental school or other departments. Individuals with an undergraduate level degree DDS can only proceed to get Masters degrees, while graduate-level degree DDS candidates may proceed to study in a PhD program.

The existing dental education was composed of preliminary curriculum focused on basic academic courses and apprentice-centered clinical education while concentrating on understanding and analyzing diagnosis and treatment of oral diseases. However, as the educational paradigm focusing on the competency of academic achievement changed, the training of professionals with integrated competencies has become an integral objective [7]. Thus, reconsideration of the changes in the new educational system was established in dental education.

The purpose of this study was to analyze the issues in potential improvements through comparison of current educational curriculums among students of a university currently operating both 3 + 4 and 4 + 4 systems. The study also aims to propose an agenda for optimal dental education system by examining the educational satisfaction and self-assessment of perceived competency among dental students.

## Materials and methods

This study was approved by the Institutional Review Board of Seoul National University School of Dentistry (protocol S-D-20190026) in accordance with the policy on research with human participants. To conduct a thorough analysis of educational satisfaction, an education system review committee was formed with four education experts with more than 10 years of teaching experience in dental education.

### Questionnaire and variables

The questionnaire of this study was developed and validated by four education experts including one clinical professor, one basic science professor, and two professors majoring in medical education. The questionnaire comprised four domains and 65 items as follows: respondent characteristics (5 items), satisfaction with undergraduate courses by subjects (24 items), the interconnection of undergraduate courses by subjects with the graduate-level DDS curriculum (24 items), and self-assessed core competency for dental graduates (12 items). The items were assessed using a 5-point Likert scale that allowed respondents to indicate their positive-to-negative strength of agreement regarding each statement.

University education satisfaction is one of the main outcome variables, consisting of overall satisfaction with general undergraduate learning experience and course satisfaction by major undergraduate subjects. Overall interconnection denotes the overall degree of connectivity between undergraduate majors and graduate DDS curriculum, and course connection points out how closely linked undergraduate specific subjects are with graduate DDS curriculum from student perspectives. Competency means self-assessed core competency for dental graduates.

### Data collection

A total of 252 students responded to the survey, among which 109 students were in the 3 + 4 system and 143 are in the 4 + 4 system. Data collection was conducted by researchers with no interest in this research to de-identify personal information so that the professors in charge of the subject would not harm students, the vulnerable subject group. In case of no response, the entry was excluded from analysis, which required scaling.

### Statistical analysis

The data analysis was conducted using SPSS version 23 (SPSS Inc., Chicago, IL, US), which involved descriptive statistics, calculations for the means, standard deviations (*SDs*). Cronbach’s correlation analysis was conducted for each evaluation item, and the average of each item score was calculated except for items that greatly reduced the internal consistency of each item. The sum of the representative values of each item was taken as the satisfaction point. The satisfaction level was analyzed using independent t-test depending on the items of the two samples, and *P*-values < 0.05 were considered to indicate statistically significant differences.

## Results

Descriptive statistics are presented in Table 1. The majority of students were male in both the 3 + 4 and 4 + 4 systems, at 53.7 and 63.1%, respectively. The 3 + 4 systems were only composed of graduates from an undergraduate dental college because it is a single curriculum, similar to the fast track in US dental schools. Among the students in the 4 + 4 system, 38.5% had majored in engineering, followed by 22.4% in natural sciences, 18.9% in dentistry, 7.7% in agriculture, 5.6% in life sciences, 2.8% in pharmacy, and 2.1% in education.

**Table 1:** Descriptive statistics of Korean dental students enrolled for each school system

Classification	3 + 4 system		4 + 4 system	
	Frequency (*N* = 109)	%	Frequency (*N* = 143)	%
**Gender**				
Male	58	53.7	89	62.2
Female	50	46.3	54	37.8
Missing	1			
**Undergrad Major**				
Dentistry	109	100	27	18.9
Engineering			55	38.5
Natural Science			32	22.4
Agriculture			11	7.7
Life Science			8	5.6
Pharmacy			4	2.8
Education			3	2.1
Liberal Studies			2	1.4
Humanities			1	0.6

As suggested in Table 2, a survey was conducted to compare educational satisfaction between students in the 3 + 4 and 4 + 4 programs, and the answers differed in each area due to the students’ distinct undergraduate backgrounds. The overall satisfaction level of the bachelor’s education was significantly higher among students in the 4 + 4 system than among those in the 3 + 4 system (*P* = 0.003). According to the academic areas, the difference between the two groups was noticeably significant among those who majored in engineering, medicine, convergence studies, and mathematical statistics. In particular, 4 + 4 students who majored in natural science, engineering, and mathematical statistics were more satisfied with their undergraduate learning experience, while 3 + 4 students with experience in the fields of medicine, social science, and convergence studies in displayed significantly high satisfaction levels.

**Table 2:** Undergrad Education satisfaction comparison of 3 + 4 and 4 + 4 systems for Korean dental students

Undergrad Education		3 + 4		4 + 4		t	*P*
		M	SD	M	SD		
Overall Satisfaction		3.52 (*n* = 107)	0.793	3.83 (*n* = 143)	0.831	−2.965	0.003
Course Satisfaction	Natural Sciences	3.51 (*n* = 99)	1.073	3.78 (*n* = 132)	0.832	−2.120	0.035
	Engineering	3.02 (*n* = 108)	0.867	3.56 (*n* = 133)	0.949	−4.590	< 0.001
	Medicine	3.94 (*n* = 108)	0.852	3.18 (*n* = 117)	0.952	5.223	< 0.001
	Humanities	3.61 (*n* = 109)	0.720	3.41 (*n* = 120)	0.886	1.937	0.054
	Social Sciences	3.47 (*n* = 109)	0.760	3.25 (n = 120)	0.901	2.023	0.044
	Convergence Studies	3.59 (n = 109)	1.020	3.08 (*n* = 137)	0.802	4.206	< 0.001
	Statistics	2.99 (*n* = 109)	1.084	3.64 (*n* = 137)	0.855	−5.269	< 0.001
	Life Science	3.59 (*n* = 105)	0.958	3.65 (*n* = 140)	0.944	−0.485	0.628
	Earth Science	2.69 (*n* = 108)	1.116	2.80 (*n* = 129)	1.018	− 0.810	0.419

Data are shown as mean values (M) and standard deviations (SD)

In Table 3, the degree of interconnectedness between the DDS graduate curriculum and the undergraduate experience of both systems was compared. Since the students had different undergraduate educational backgrounds, the number of responses w varied by category. Significant differences were not observed in the connection between the overall undergraduate education experience and the DDS graduate curriculum; however, a detailed examination of undergraduate education by field of study between the two groups revealed a significant difference among students in the fields of natural sciences.

**Table 3:** Comparison of 3 + 4 or 4 + 4 systems in interconnection degree b/n undergrad and DDS curriculums for Korean dental students

Undergrad/DDS Curriculum	3 + 4		4 + 4		t	*P*
	M	SD	M	SD		
Overall Interconnection	3.14 (*n* = 104)	0.989	3.27 (*n* = 143)	1027	−0.932	0.352
Degree of connection by subjects						
Natural Sciences	2.98 (*n* = 98)	1.074	3.68 (*n* = 134)	.906	−5.367	< 0.001
Engineering	2.79 (*n* = 82)	0.923	3.01 (*n* = 132)	.961	−1.660	0.098
Medicine	3.93 (*n* = 83)	0.894	3.45 (*n* = 117)	1.030	3.390	0.001
Humanities	2.98 (*n* = 109)	0.953	2.59 (*n* = 123)	1.039	2.952	0.003
Social Sciences	2.88 (*n* = 98)	0.900	2.55 (n = 123)	1.034	2.481	0.014
Convergence Studies	2.72 (*n* = 98)	1.138	2.54 (*n* = 127)	0.904	1.293	0.198
Statistics	3.13 (*n* = 109)	1.187	2.69 (*n* = 135)	0.973	3.178	0.002
Life Sciences	3.96 (*n* = 83)	0.865	3.80 (*n* = 137)	0.940	1.325	0.187
Earth Sciences	2.42 (*n* = 97)	1.126	2.17 (*n* = 127)	0.977	1.772	0.780

Data are shown as mean values (M) and standard deviations (SD)

In particular, the 4 + 4 students recognized that undergraduate learning experiences in natural sciences were more connected to the DDS graduate curriculum than did the 3 + 4 students, while the 3 + 4 students recognized that studies in medicine, humanities, social sciences and mathematical statistics were closely interconnected to the graduate-level DDS curriculum.

While there were differences in the students’ perception of core competency for dental graduates with respect to their own performance, almost no statistically significant difference in the self-assessed competency between 3 + 4 and 4 + 4 students was observed (see Table 4). Although statistical differences were not observed, 3 + 4 students generally reported lower self-perception of competency than did 4 + 4 students.

**Table 4:** Comparison of Korean dental students’ self-assessed competency for dental graduates b/n 3 + 4 and 4 + 4 systems

Core Competency	3 + 4 (*n* = 82)		4 + 4 (*n* = 141)		t	*P*
	M	SD	M	SD		
Professionalism	3.73	0.802	3.90	0.793	−1.537	0.126
Interpersonal Skills	3.77	0.790	3.86	0.786	−0.832	0.407
Critical Thinking	3.77	0.634	3.77	0.829	−0.064	0.949
Clinical Information	3.44	0.818	3.57	0.862	−1.119	0.264
Treatment Plan	3.40	0.799	3.56	0.777	−1.414	0.159
Oral Health Rebuilding	3.32	0.915	3.55	0.835	−1.931	0.055
Health Promotion	3.51	0.707	3.70	0.788	−1.823	0.070

Data are shown as mean values (M) and standard deviations (SD)

## Discussion

Recently, focus has shifted to improving the quality of university education services based on a student-centered paradigm. Universities must improve their accountability by understanding student needs, perspectives, and values, and altering university management and curriculum as needed [8]. In this regard, student satisfaction surveys can be used as a basis to systematically analyze student perceptions for university education [9]. In addition, surveys of achievement levels perceived by students, including learners’ attitudes toward learning, are important factors in examining the effectiveness of education [10]. Based on the results of this survey examining the satisfaction, educational linkage, and self-perceived competence of students enrolled in 3 + 4 or 4 + 4 dental education system, the validity of the school system was analyzed and the ways to improve dental education were examined.

The 4 + 4 students reported better educational satisfaction than did the 3 + 4 students. The difference in satisfaction between the two systems must be considered in conjunction with the selection and curriculum formation. The 4 + 4 students likely had concrete plans for their career and prepared specific goals during their career planning process because they go through another competitive admission process to get into the dental school graduate program. In contrast, the 3 + 4 s students are directly enrolled into the program from high school and stay until they receive DDS at the end of 7 years of coursework, and thus experience relatively fewer concerns about dental career and aptitude.

According to previous studies, motivation for career and learning in pre-dental undergraduate students is generally low [11, 12]. Students’ professional motivations vary by developmental stage during the education curriculum. In the career choice model, the period of career and job choices are viewed after age 18 or 25 [13]. Academic enthusiasm or self-directedness exhibited by motivated students is a factor when choosing a curriculum [14]. However, these criteria may change depending on when students experience strong professional motivation, which could be after high school or after undergraduate education. Personal views and concepts regarding the profession are among the major influencing factors for choosing dentistry as a career [15].

The Flexner report in 1910 also suggested that a four-year medical curriculum is ideal and claimed that at least 2 years of basic science must be studied in college prior to studying medicine [16, 17]. In line with this argument, from the 1920s, 92% of US medical schools selected applicants with basic science skills [18]. This is the logic behind the development of the 4 + 4 system, the current medical school system in the US. An interesting result of this study is that 3 + 4 students displayed higher satisfaction with social sciences and interdisciplinary studies, while 4 + 4 students revealed higher satisfaction with the natural sciences, engineering, and mathematical statistics. The 3 + 4 system is designed for early exposure to courses that are highly relevant to dentistry majors, such as dentistry, genetics, and cellular molecular biology. In addition, the ratio of multidisciplinary subjects is high to improve problem-solving ability for various social issues required by project-based learning. These project-based approaches can help contextualize scientific research in dental curriculums [19].

On the other hand, it can be inferred that most of the 4 + 4 students received their bachelors’ degrees in natural science and engineering and that they found themselves more satisfied with the major-related courses that are directly related with the DDS curriculum. This is in line with the primary intentions of the US medical education reform based on the Flexner. To improve the integrity of the 7-year curriculum with a 3 + 4 system, preparation for the basic subjects must be systematically accompanied, as in the previous studies in which the undergraduate course is regarded as a period for building a foundation of basic courses in medical education [20]. In addition, as suggested by Dienstag, the undergraduate education should not only be a mere preparation course for basic medical education but should also include creative educational experiences that can expand intellectual exploration and expose students to a broader liberal arts education [21]. Thus, it is necessary to monitor the effects of different education systems and previous education experiences on the academic achievements after enrolling in a professional graduate dental school.

Globally, the most common goals in dental education are to cultivate excellent dental clinicians, foster dental scientists, and promote leaders in various fields [22, 23]. Although the 3 + 4 and 4 + 4 systems all aim to educate dental clinicians, the 4 + 4 system is more suitable for fostering academic leaders in convergent disciplines, especially in terms of encouraging students from different undergraduate backgrounds. For the successful operation of the 3 + 4 system, more emphasis on clinical, research, and leadership pathways could be considered. By selecting a career path from the entrance stage with continuous monitoring, it is possible to ensure competence in specialized areas that could be evaluated at graduation.

Acknowledging that an institution’s educational objective is the primary criteria for developing a curriculum, an individual dental school, which selects students in 3 + 4 and 4 + 4 systems, may experience difficulties in setting integrated educational goals [24]. Educational objectives should be established to accommodate each stage of dental education, and the curriculum must be designed accordingly. In the 3 + 4 system, which has a relatively low level of satisfaction, it is urgent to clearly define and reflect the core competencies and educational achievements in the curriculum that must be reached during the first 3 years before entering the later 4 years of graduate school.

On the other hand, undergraduate students entering professional graduate school through the 4 + 4 system require strengthening in the areas of medical humanities and social dentistry. To this end, a dedicated department must be established to develop and assess a curriculum that incorporates humanity and social dentistry subjects with fundamental features of dental education. Dental student’s exposure to humanities is recognized as an important element in the attitudes of dental practitioners [25]. However, training methods to achieve these competencies have not yet been clearly defined, and outcome measurements remain elusive. Since the areas in logical reasoning, data analysis, and situation analysis are evaluated by the Public Service Aptitude Test in Korea, it is also possible to consider the adaptation of various methods for evaluating educational performance of the humanities and social dentistry. In addition, this study suggests that the entire dental college curriculum should be integrated with the consideration of connectivity. The basic science courses taught in the undergraduate curriculum and the clinical practice courses in the graduate DDS curriculum should be linked seamlessly so that students can experience the entire course of evidence-based clinical care.

Although this study is mainly concerned with the learner’s perspective, the educational system must also be noted in consideration with the social context of educational institutions. A variety of factors, such as changes in the academic system, the development of technology, and needs of the public and the community, influence the decision of the appropriate educational system, especially, the education system of health professionals [26]. The dental education systems in North America and Europe, which have been the major touchstones of dental education, are also changing constantly to fulfill societal expectations. Continuous research on the duration and curriculum of dental training that are required to attain competencies for dentist must be maintained [27].

## Supplementary Information

**Additional file 1.**

## Data Availability

The datasets used and/or analysed during the current study are not publicly available due to limitations of ethical approval involving the student data and anonymity but are available from the corresponding author on reasonable request.

## Abbreviations

US: United States
UK: United Kingdom
DDS: Doctor of Dental Surgery Degree
