# Supplementary material for: Dental students’ self-evaluation comparison between dual dental education systems in Korea
Source: BMC Med Educ. 2022 Jun 6;22:433. doi: 10.1186/s12909-022-03504-6 (PMC9171986; doi:10.1186/s12909-022-03504-6)
Supplement: Supplementary file 1 — Additional file 1. [file 12909_2022_3504_MOESM1_ESM.docx]

**Supplementary Table 1. Pre-dental(3-year) curriculum of a Korean dental school in 3 + 4 system**

|  | **First Year** | **Second Year** | **Third Year** |
| --- | --- | --- | --- |
| **1^st^ Spring Semester** | Freshmen Seminar  Academic Writing  Foreign Languages  Mathematics  Electives | Cellular and Molecular Biology  Science and Ethics  Dentistry in History and Culture  *Project-based Learning 2*  Electives | Immunology  *Independent Dental Research 1*  Electives |
| **2^nd^**  **Fall Semester** | Physics  Chemistry  Biology  Medical Statistics  *Project-based Learning 1*  Electives | Genetics  Biodiversity & Global Environment  Dental Communication  Medical Data Management  *Project-based Learning 3*  Electives | Introduction to Dentistry  Dental Engineering  *Independent Dental Research 2*  Electives |

*Note. Project-based Learning* is a learner-centered program where students are encouraged to deal with multidisciplinary subjects to examine dental issues and propose the alternatives to them. *Independent Dental Research* allows students to participate in the scientific research as an intern in different labs operated by dental faculty members.
